# Infiltrating stromal immune cells in inflammatory breast cancer are associated with an improved outcome and increased PD-L1 expression

**DOI:** 10.1186/s13058-019-1108-1

**Published:** 2019-02-18

**Authors:** C. Van Berckelaer, C. Rypens, P. van Dam, L. Pouillon, M. Parizel, K. A. Schats, M. Kockx, W. A. A. Tjalma, P. Vermeulen, S. van Laere, F. Bertucci, C. Colpaert, L. Dirix

**Affiliations:** 1 Translational Cancer Research Unit, GZA Hospitals & CORE, MIPRO, University of Antwerp, Antwerp, Belgium; 2 Multidisciplinary Breast Clinic, Unit Gynaecologic Oncology, Antwerp University Hospital (UZA), Edegem, Belgium; 3 Department of Medical Oncology, GZA Hospitals Sint-Augustinus, Antwerp, Belgium; 4 Department of Pathology, Antwerp University Hospital (UZA), Edegem, Belgium; 5 HistoGeneX, Antwerp, Belgium; 6 Predictive Oncology team, Centre de Recherche en Cancérologie de Marseille (CRCM), Inserm, CNRS, Aix-Marseille Université, Institut Paoli-Calmettes, Marseille, France; 7 Department of Pathology, GZA Hospitals Sint-Augustinus, Antwerp, Belgium

**Keywords:** Inflammatory breast cancer (IBC), Immune response, Programmed death-ligand 1 (PD-L1), Stromal tumour-infiltrating lymphocytes (sTIL), Immune checkpoint modulators

## Abstract

**Background:**

Inflammatory breast cancer (IBC) is a rare and rapidly progressive form of invasive breast cancer. The aim of this study was to explore the clinical evolution, stromal tumour-infiltrating lymphocytes (sTIL) infiltration and programmed death-ligand 1 (PD-L1) expression in a large IBC cohort.

**Patients and methods:**

Data were collected prospectively from patients with IBC as part of an international collaborative effort since 1996. In total, 143 patients with IBC starting treatment between June 1996 and December 2016 were included. Clinicopathological variables were collected, and sTIL were scored by two pathologists on standard H&E stained sections. PD-L1 expression was assessed using a validated PD-L1 (SP142) assay. A validation cohort of 64 patients with IBC was used to test our findings.

**Results:**

Survival outcomes of IBC remained poor with a 5-year overall survival (OS) of 45.6%. OS was significantly better in patients with primary non-metastatic disease who received taxane-containing (neo)adjuvant therapy (*P =* 0.01), had a hormone receptor-positive tumour (*P =* 0.001) and had lower cN stage at diagnosis (*P =* 0.001). PD-L1 positivity on immune cells (42.9%) was higher in IBC than in non-IBC in both our patient samples and the validation cohort. Furthermore, PD-L1 expression predicted pCR (*P =* 0.002) and correlated with sTIL infiltration (*P <* 0.001). sTIL infiltration of more than 10% of the stroma was a significant predictor of improved OS (HR 0.47, 95% CI 0.27–0.81, *P =* 0.006) in a multivariate model.

**Conclusions:**

IBC is characterised by poor survival and high PD-L1 immunoreactivity on sTIL. This suggests a role for PD1/PD-L1 inhibitors in the treatment of IBC. Furthermore, we showed that PD-L1 expression predicts response to neo-adjuvant therapy and that sTIL have prognostic significance in IBC.

**Electronic supplementary material:**

The online version of this article (10.1186/s13058-019-1108-1) contains supplementary material, which is available to authorized users.

## Background

Inflammatory breast cancer (IBC) is an uncommon and aggressive form of breast cancer, characterised by rapid progression and a high risk of lymph node involvement or distant metastasis at diagnosis [1]. IBC accounts for a disproportional fraction of breast cancer-related mortality and tends to affect mainly younger women. This results in a significant loss of life years [2]. Furthermore, the incidence appears to be increasing [3] and IBC-specific therapies are lacking.

An international expert panel on IBC agreed that the diagnosis of IBC remains based on clinical features (Additional file 1: Table S1), and all patients should be treated with neo-adjuvant anthracycline- and taxane-based chemotherapy (NACT), radical modified mastectomy in non-metastatic patients and radiotherapy including the supraclavicular regions and internal mammary lymph nodes [4]. Hormonal or targeted therapy against HER2 is offered if indicated. Notwithstanding this aggressive treatment, the overall survival (OS) remains lower than in non-inflammatory breast cancer (nIBC) [5]. Therefore, further research is warranted to unravel the molecular biology of IBC. In this context, the World IBC Consortium (WIBCC) reported a 79-gene signature that discriminates between IBC and nIBC patient samples, indicating a distinct molecular basis for IBC [6]. Translating this gene signature into molecular concepts suggested that the biology of IBC is characterised by an altered TGF-β pathway and immune response program. Thus, a specific tumour immune microenvironment with infiltrating immune cells might contribute to the unique biological features associated with IBC [6, 7]. Other research groups have also underlined the importance of the tumour stroma in IBC [8, 9]. The invasion of the tumour stroma by stromal tumour-infiltrating lymphocytes (sTIL) is often associated with a better prognosis in ER-negative, more proliferative subtypes of nIBC [10]. Since IBC is characterised by an increased mutational load, this might lead to increased tumour antigen-based attraction of cytotoxic T cells [11].

Programmed death-ligand 1 (PD-L1) is an immune checkpoint molecule that can be expressed on both infiltrating immune cells and tumour cells. While PD-L1 positivity in breast cancer was associated with worse survival and adverse clinicopathological features [11], an improved response to therapy and better outcome in triple-negative breast cancer (TNBC) was also seen [12, 13]. An upregulation of PD-L1, as a marker of active immune response against TNBC might be a possible explanation [14]. Bertucci et al. showed on mRNA level that PD-L1 expression in IBC was associated with an improved response to NACT and that this effect was enhanced compared to nIBC [15]. In a large IHC study, pCR was also more common in patients with PD-L1 expression (on tumour cells) and an association with lymph node status and CD20+ TILs was seen [16]. However, the prognostic effect of PD-L1 in IBC remains ambiguous as different studies generated conflicting results [15–19] (Table 1).

**Table 1:** Overview of all PD-L1 studies in IBC and their key findings. *NACT* neo-adjuvant chemotherapy, *IHC* immunohistochemistry, *TMA* tissue microarray, *TC* tumour cell, *IC* immune cell, *N+* lymph node-positive disease, *pCR* pathological complete response, *NR* not reported, *OS* overall survival, *DFS* disease-free survival, *sTIL* stromal tumour-infiltrating lymphocytes, *ER* oestrogen receptor, *T/NB* tumour/normal breast ratio, *TN* triple negative

First author (year, country)	No.	Population	Detection method	AB clone	Cutoff for PD-L1+	Positivity rate	Associated clinical and pathological variables	Associated outcome variables
He et al., [17] (2018, USA)	68	Post NACT TN: 19/65	IHC TMA [3]	28-8	≥ 1% TC	25/68 (36.8%)	/	Worse OS (*P =* 0.042)
Arias-Pulido et al. [16] (2018, Algeria)	221	Pre NACT TN: 44/221	IHC TMA [2]	SP142	≥ 5% TC and IC	TC: 18/221 (8.1%) IC: 146/221 (66.1%)	TC: N+, CD20+ TIL, pCR IC: grade 3, CD20+ TIL	IC: better DFS (*P =* 0.035)
Reddy et al. [18] (2017, USA)	14	Pre NACT TN: -	IHC Biopsy	NR	NR (TC)	3/14 (21.4%)	NR	NR
Hamm et al. [19] (2016, USA)	12	Pre NACT TN: -	IHC Biopsy	E1LN3	H-score (TC and IC)	TC: 4/12 (33.3%) IC: 8/12 (66.7%)	NR	NR
Bertucci et al. [15] (2015, France)	112	Pre NACT TN: 28/112	mRNA	/	T/NB ≥ 2	42/112 (37.5%)	ER negativity, basal and HER2+ subtype, cytotoxic T cell response, pCR	/
Discovery cohort (Belgium)	105	Pre NACT TN: 23/105	IHC Biopsy	SP142	≥ 1% tumour area (TC and IC)	TC: 2/105 (1.9%) IC: 45/105 (42.9%)	IC: sTIL, pCR	/
Validation cohort (France)	62	Pre NACT TN: 12/62	IHC Biopsy	SP142	≥ 1% tumour area (TC and IC)	TC: 0/62 (0%) IC: 24/62 (38.7%)	IC: sTIL	/

It is still unclear if and how the immune response is able to influence the IBC phenotype with its fulminant local progression, typical tumour emboli formation and rapid metastasis. The aim of this study was to explore sTIL infiltration, PD-L1 expression and the clinical evolution in a cohort of 143 IBC patients diagnosed and treated in Antwerp, Belgium, and in a validation cohort of 64 patients from Marseille, France.

## Methods

### Patient selection

We analysed clinical data and tumour specimens from 143 consecutive patients with IBC who had their initial diagnosis and complete treatment at the GZA Hospital Sint-Augustinus or the Antwerp University Hospital between June 1, 1996, and December 31, 2016. All cases were diagnosed based on the clinical IBC definition (Additional file 1: Table S1) and were pathologically confirmed as invasive carcinoma. Clinicopathological variables of all patients were collected from in-hospital medical records. Estrogen (ER) and progesterone receptor (PgR) expression had been assessed in the pathology department using validated immunohistochemical tests and defined as positive if Allred score ≥ 3/8. Tumours were considered HER2-positive when a fluorescence in situ hybridisation (FISH) test documented amplification. The absence of residual invasive carcinoma in the resected breast specimen and in all sampled regional lymph nodes after completion of NACT was defined as pathological complete response (pCR). Pre-treatment tumour tissue samples (34% core needle and 66% open biopsy samples) were analysed for sTIL and PD-L1. Subtype-matched nIBC patients (*N =* 142), diagnosed in Sint-Augustinus in 2006, were selected as a control group for the comparison of sTIL and PD-L1 scoring between IBC and nIBC. An extra cohort of 64 IBC (M0—stage III) patients diagnosed and treated in Marseille, France (Institut Paoli-Calmettes), was used as a validation cohort.

### Stromal tumour-infiltrating lymphocytes (sTIL)

TIL scoring was performed on haematoxylin and eosin (H&E) stained 5-μm sections of formalin-fixed paraffin-embedded (FFPE) pre-treatment tumour tissue by two different researchers (CC and MP) according to the recommendations by the International TILs Working Group [20]. Given the specific pathology of IBC with often small and dispersed tumour cell nests, TILs were reported for the stromal compartment (% stromal TILs, sTIL) in all areas containing invasive tumour cells on the H&E slide containing the most invasive tumour. The mean scores of both pathologists were used both as continuous and categorical variables: < 10% (category 1), ≥ 10–40% (category 2), and ≥ 40% (category 3). The interclass correlation coefficient (ICC) (two-way, agreement model) was 0.738 (95%CI 0.628–0.819, *P* <  0.001) showing a substantial agreement.

### PD-L1

PD-L1 expression was assessed on 5-μm FFPE slides using a validated PD-L1 assay (clone SP142, Ventana Benchmark) on the tumour (TC) and infiltrating immune cells (IC). The staining protocol was optimised independently and previously described [21]. Scoring was done by two different researchers (CC and CVB) and based on the percentage of the tumour area that was occupied by PD-L1+ immune cells or the percentage of PD-L1+ tumour cells. A score of 0 (= PD-L1−), 1, 2 or 3 was assigned for < 1%, ≥ 1% but < 5%, ≥ 5% but < 10% or ≥ 10% PD-L1-positive cells per tumour area, respectively [22]. These cutoffs were used for both tumour and immune cells (ICC 0.854, 95%CI 0.792–0.819, *P* < 0.001), and in case of a discrepancy between the researchers, a consensus score was determined.

### Statistical analysis

Data were analysed using R studio (Version 1.1.414). Cases with missing data were maintained in the database but excluded from the statistical analyses on a per test basis. Pearson chi^2^ test (categorical variables) and ANOVA (continuous variables) were used to assess the relationship between the different parameters. Significant parameters were included in a multivariate regression model. Evaluated survival endpoints were recurrence-free survival (RFS), distant metastasis-free survival (DMFS) and overall survival (OS). Survival data were last updated on January 1, 2018. Survival curves were calculated with Kaplan-Meier estimates and compared using the log-rank test. Significant clinicopathological variables were included in a multivariate Cox proportional hazards model. *P* values were calculated two-sided and considered statistically significant when < 0.05.

## Results

### Clinicopathological characteristics

**Fig. 1:**
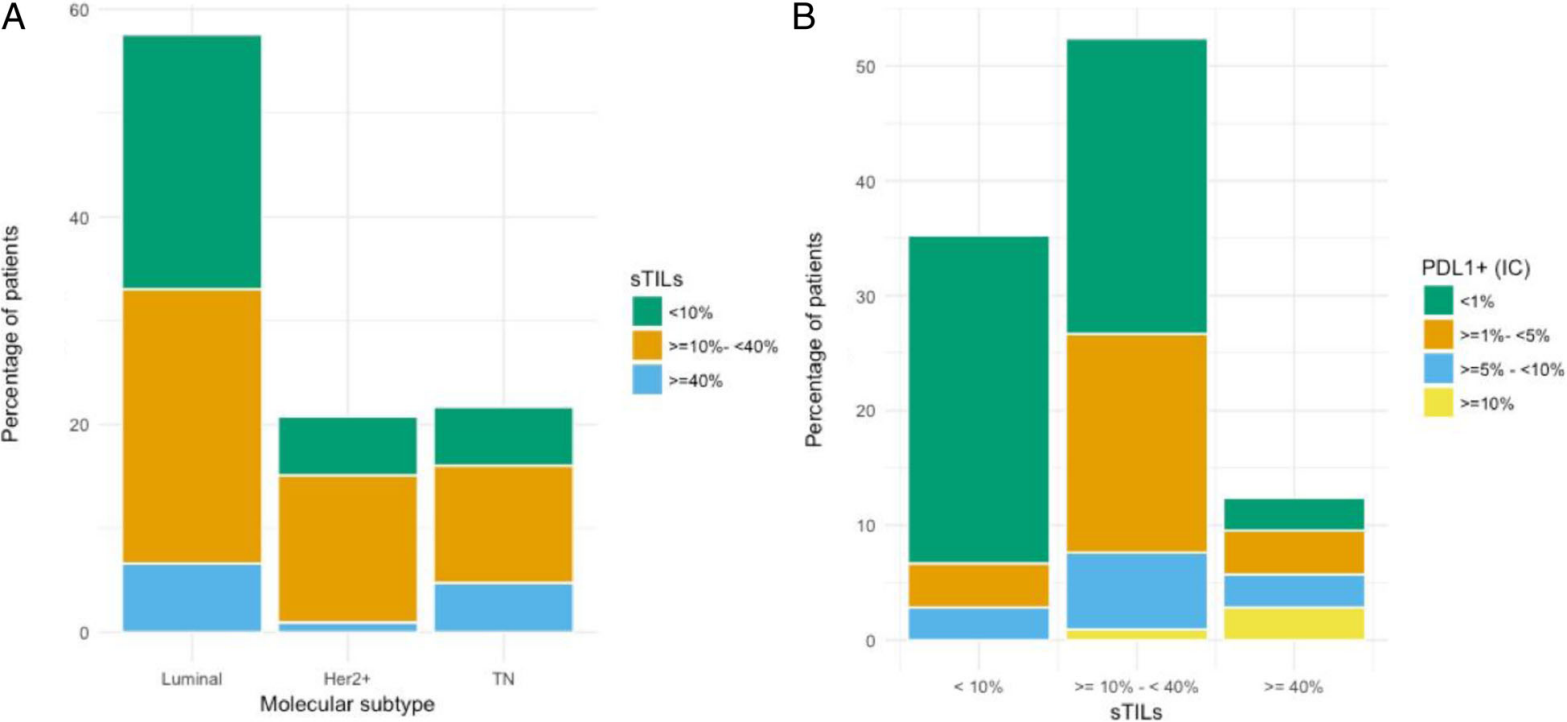
**a** Distribution of sTIL scores in the different molecular subtypes (*N =* 138). Luminal (HR+) 55.1% (*N =* 76), HER2+ 21.7% (*N =* 30), TN (HR-Her2−) 23.2% (*N =* 32). **b** Distribution of PD-L1 positivity in the categories of sTIL scores (*N =* 106): 35.8% (*N =* 38), 51.9% (*N =* 54), 12.3% (*N =* 13). There is a strong correlation with PD-L1 immunoreactivity on immune cells (*χ*^2^  = 28.9, *P <* 0.001)

Overall, 143 patients were included and clinicopathological characteristics are provided in Table 2. Most tumours were invasive ductal adenocarcinomas (*N =* 134, 94.4%) and poorly differentiated (*N =* 94, 71.2%). Half of the patients presented with an ER-positive tumour (*N =* 74, 52.5%), and 61 (44.5%) patients had a HER2-positive tumour (Fig. 1a). At the time of diagnosis, 103 (72.0%) patients had node-positive disease and 40 (28.0%) patients presented with primary metastatic disease. From the patients without metastatic disease at diagnosis, 91 patients (88.3%) completed NACT. Most of these patients underwent radical mastectomy (83/91, 91.2%) followed by radiotherapy (84/91, 92.3%). Considering that systemic therapies have changed during this study time interval, only 49 out of 78 patients (62.8%) with HR-positive cancer completed adjuvant hormonal therapy (23 patients received tamoxifen and 26 patients an aromatase inhibitor). Less than half of the patients with HER2+ cancer received targeted therapy (27/62, 43.5%). An anthracycline-based regimen (78/91, 85.7%) combined with a taxane (74/91, 81.3%) was the most common type of NACT (Additional file 1: Figure S1). Almost a quarter of the patients that underwent a mastectomy after completing NACT (20/82, 24.4%) achieved a pCR, but no clinicopathological variable predicted pCR.

**Table 2:** Patient and tumour characteristics for the IBC series, (*n*) = number of patients. *ER* oestrogen receptor, *PgR* progesterone receptor, *HR* hormone receptor

Mean age (143)	60.1 years (25.7–91.2 years)	
Mean sTIL score (106)	17.63%, 95% CI 15.00–20.26%	
Menopausal status (142)	Premenopausal	42 (29.4%)
	Postmenopausal	101 (70.6%)
cN stage (142)	0	6 (4.2%)
	1	53 (37.9%)
	2	52 (37.1%)
	3	29 (20.7%)
cM stage (143)	0	103 (72.0%)
	1	40 (28.0%)
Pathological type (142)	Ductal	134 (94.4%)
	Lobular	5 (3.5%)
	Mixed	3 (2.1%)
Differentiation (133)	Grade 1	3 (2.3%)
	Grade 2	35 (26.3%)
	Grade 3	95 (71.4%)
ER (141)	Negative	67 (47.5%)
	Positive	74 (52.5%)
PgR (141)	Negative	88 (62.4%)
	Positive	53 (37.6%)
HER2+ (139)	Negative	77 (55.4%)
	Positive	62 (44.6%)
Molecular subtype (138)	Luminal (HR+)	76 (55.1%)
	HER2+ (HR-HER2+)	30 (21.7%)
	TN (HR-HER2−)	32 (23.2%)
sTIL (106)	< 10%	38 (35.8%)
	≥ 10 to < 40%	54 (51.9%)
	≥ 40%	13 (12.3%)
PD-L1 immune cells (105)	< 1%	60 (57.1%)
	≥ 1 to < 5%	28 (26.7%)
	≥ 5 to < 10%	13 (12.4%)
	≥ 10%	4 (3.8%)

### sTIL and PD-L1 data

**Fig. 2:**
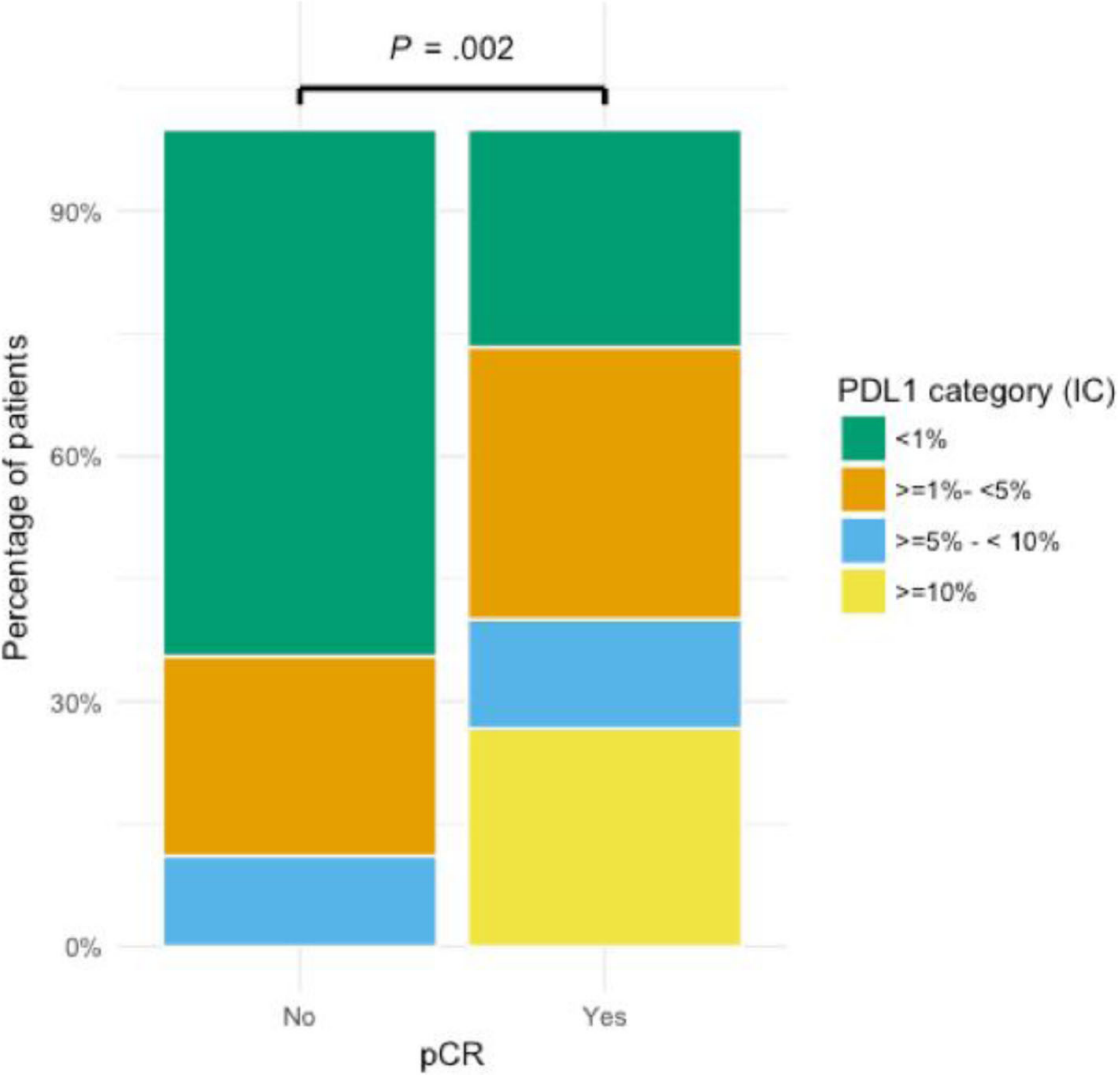
Patients that had a complete pathological response (pCR) showed more PD-L1 immunoreactivity (73.3% PD-L1+ sTIL) than patients without a complete response (36.9% PD-L1+ sTIL). *χ*^2^ = 15.3, *P =* 0.002

Mean sTIL infiltration was 17.63% (95%CI 15.00–20.26%) and median sTIL infiltration was 10.00% (Q1: 7.5%–Q3: 25.0%). Infiltration with sTIL was high in triple-negative IBC (median 17.5%, mean 22.8%, *N* = 23) compared to other IBC subtypes. However, only a trend towards significance (*P* = 0.078) was reached and no correlation with other clinicopathological parameters could be discovered. PD-L1 positivity on tumour cells was very uncommon. Only 2 out of 105 patients (1.9%) showed membranous tumour cell staining in more than 1% of the tumour cells. Immunoreactivity on the infiltrating immune cells was more frequent. In 45 out of 105 tissue samples (42.9%), at least 1% of the tumour area was occupied by PD-L1+ immune cells. There was a strong correlation between PD-L1 positivity and sTIL scores (*P <* 0.001) (Fig. 1b). A correlation between PD-L1 and differentiation (*P* = 0.01) was also observed, but unlike sTIL scores, a higher grade was not significant in the multivariate model (Additional file 1: Table S2). PD-L1 immunoreactivity of sTIL correlated with pCR (*P* = 0.002) (Fig. 2) and the mean sTIL score in patients that achieved pCR was significantly higher than in patients who did not (28.4% vs. 16.8%, *P =* 0.03). However, only PD-L1 expression remained significant in a multivariate model (OR 1.22, 95% CI 1.07–1.40, *P =* 0.005) (Additional file 1: Table S3).

**Fig. 3:**
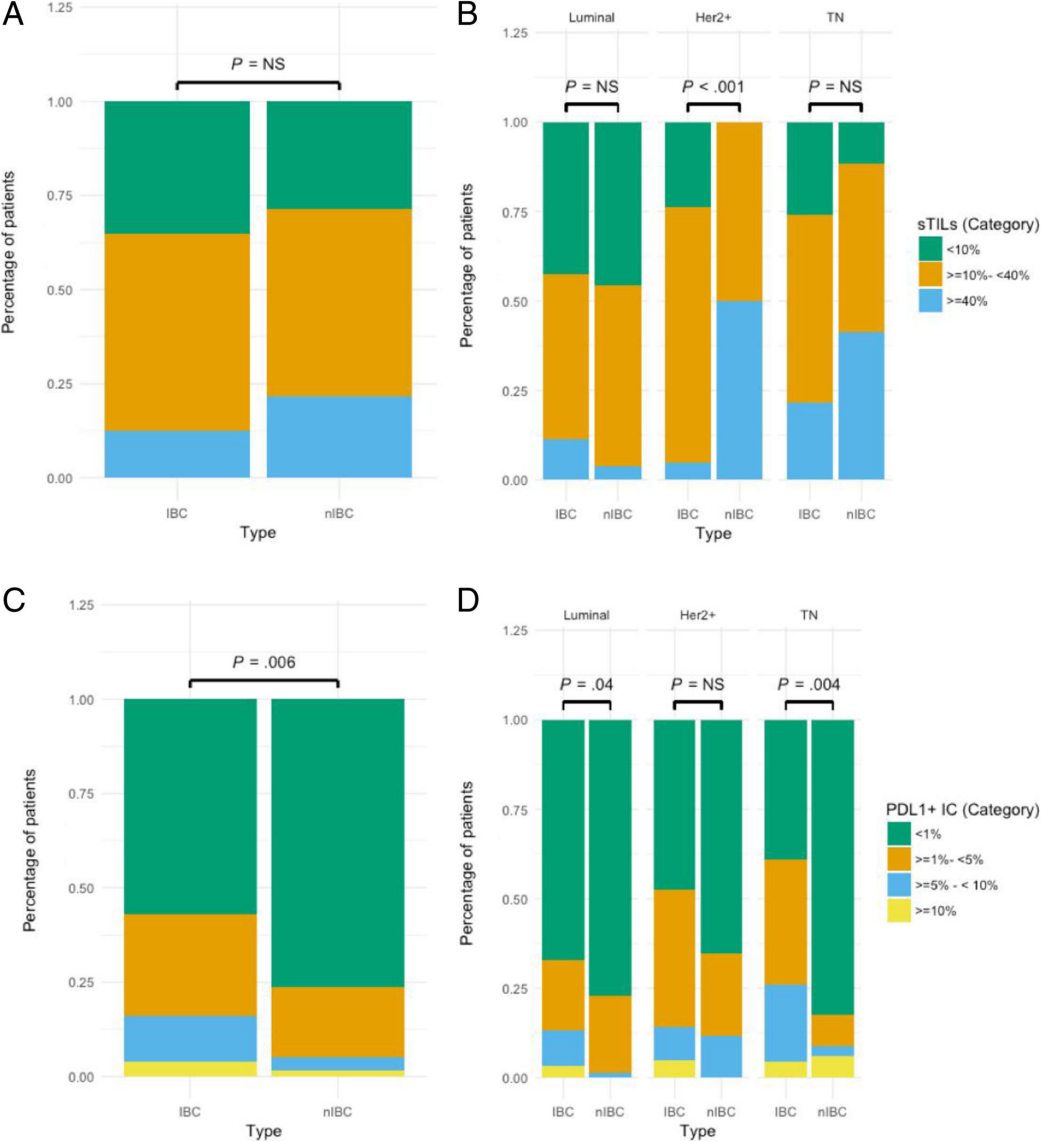
**a** In IBC, 64.2% (68/106) of the patients have ≥ 10% infiltration of the stroma with sTIL vs. 70.4% (100/142) of the nIBC patients (*P =* NS). **b** There is no significant difference in sTIL infiltration between IBC and nIBC in the luminal and TN subgroup. In the HER2+ group, 100% (16/16) of the nIBC patients have ≥ 10% infiltration of the stroma vs. 72.7% (16/22) of the IBC patients (*χ*^2^ = 16.2, *P <* 0.001). **c** In IBC, 42.9% (45/105) of the patients have ≥ 1% PD-L1 expression on the infiltrating immune cells vs. 23.7% (33/139) in nIBC (*χ*^2^ = 12.5, *P =* 0.006). **d** Luminal subtype: ≥ 1% PD-L1 expression in IBC 32.8% (20/61) vs. nIBC 22.8% (18/79) (*χ*^2^ = 8.2, *P =* 0.04). HER2+ subtype: ≥ 1% PD-L1 expression in IBC 52.4% (11/21) vs. nIBC 34.6% (9/26) (*P =* NS). TN subtype: ≥ 1% PD-L1 expression in IBC 60.9% (14/23) vs. nIBC 17.6% (6/34) (*χ*^2^ = 13.4, *P =* 0.004)

The control population of nIBC patients was age- and subtype-matched (Additional file 1: Table S4) but, in accordance with IBC biology, differed significantly for nodal status (95.7 vs. 38.6%; *P* < 0.001) and presence of metastatic disease (28.0 vs. 0.0%, *P* < 0.001) at time of diagnosis. Infiltration with sTIL was comparable between IBC and nIBC (> 10% infiltration: 64.2% in IBC vs. 70.4% in nIBC, *P =* NS). However, in the HER2+ group, TILs were significantly higher in nIBC patients (Fig. 3a, b). PD-L1 expression on sTIL was significantly more frequent in IBC (PD-L1+ samples: 42.9% in IBC vs. 23.7% in nIBC, *P =* 0.006). This difference remained significant in all but the HER2+ molecular subtype (Fig. 3c, d). In a multivariate logistic regression model, using sTIL infiltration and molecular subtype as possible confounders, patients with IBC also showed significantly more PD-L1+ immune cells (OR 2.43, 95%CI 1.19–5.04, *P* = 0.01) compared to nIBC patients.

### Survival data

The median time to disease recurrence in patients with initially localised disease was 3.50 years (95%CI 2.02–11.45 years) (Additional file 1: Figure S2A). Of the 52 patients that relapsed during the study interval, 71.2% (*N =* 42) presented with distant metastasis as the first site of relapse. Brain metastases occurred in 21 patients (40.4%) initially having local disease that developed distant disease (Additional file 1: Table S5). Brain-only relapse was observed in 9 patients (17.3%). In the total IBC population, the 5-year OS was 45.7%. Median OS was 8.92 years (95%CI 4.79– / years) in patients with initially localised disease and 2.38 years (95%CI 1.45–3.2 years) in patients with primary metastatic disease (Additional file 1: Figure S2B).

**Fig. 4:**
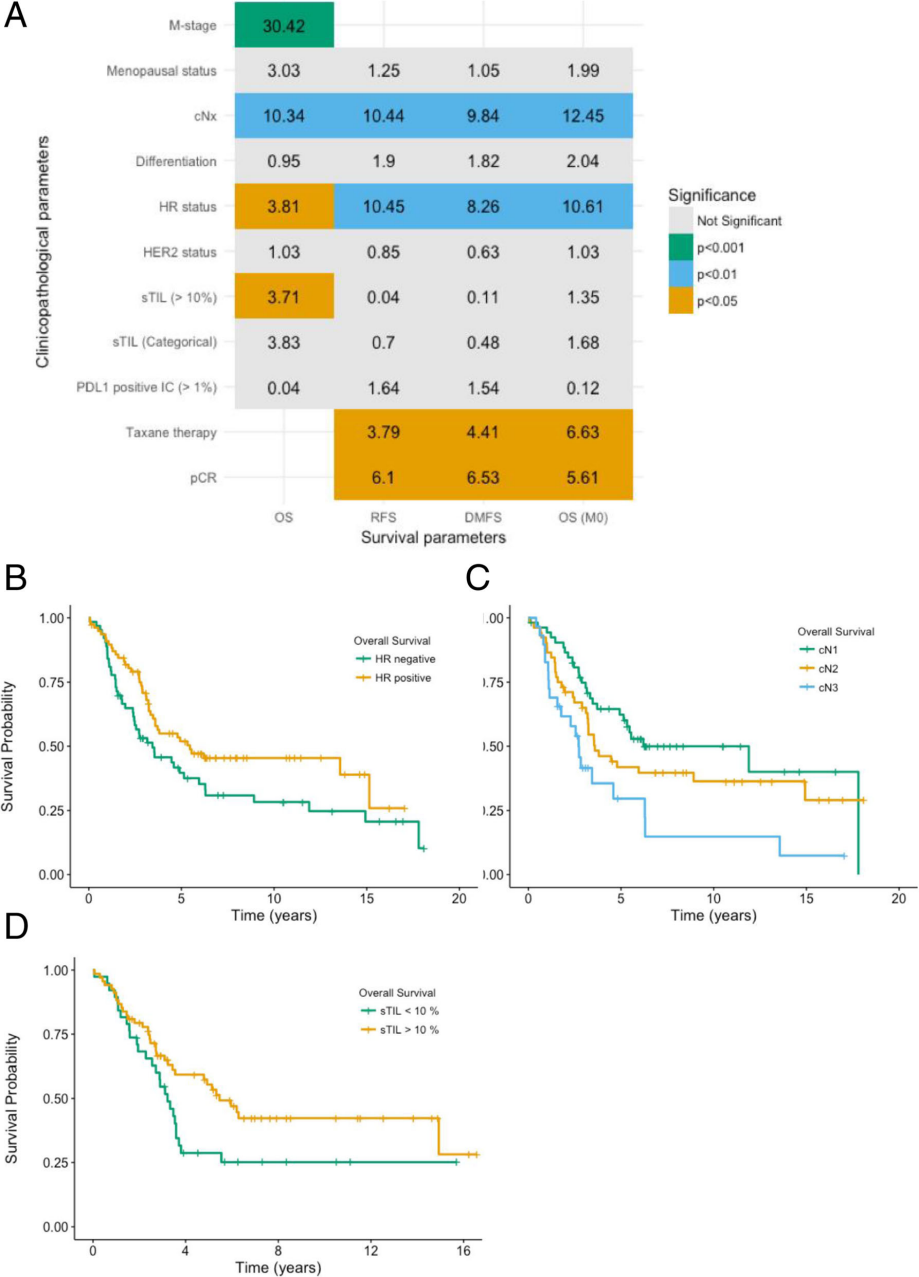
**a** Overview of all clinicopathological values with their *χ*^2^ value in the table. Compared using the log-rank test, with associated significance levels. **b–d** Kaplan-Meier curves of significant prognostic variables for OS. **b** Hormone receptor status, 5-year OS 51.6% (HR+) vs. 39.6% (HR−), *P =* 0.05. **c** Clinical nodal status, 5-year OS 62.3% (N1) vs. 41.8% (N2) vs. 29.7% (N3), *P =* 0.003. **d** sTIL score, 5-year OS 55.4% (> 10%) vs. 28.7% (< 10%), *P =* 0.05

A number of clinicopathological parameters were associated with a better prognostic outcome. A summary and corresponding OS curves are shown in Fig. 4 (curves for DMFS and RFS can be found in Additional file 1: Figures S3 and S4). A positive HR status was prognostic for a better OS in the total study population (*P =* 0.05) and was also associated with a longer RFS (*P =* 0.001) and DMFS (*P =* 0.004). Patients with HER2-positive tumours showed no significant differences in OS, RFS and DMFS when compared to patients with HER2-negative tumours. However, our data suggest that treatment with trastuzumab is associated with an improved outcome (*N =* 52, 5-year OS in HER2+ patients: 66.2% (with therapy) vs. 43.9% (without therapy), *P =* 0.04, Additional file 1: Figure S5). Other prognostic parameters were nodal disease at the moment of diagnosis, receiving taxane-containing neo-adjuvant regimens and achieving pCR after NACT. Finally, sTIL infiltration above 10% (i.e., above the median sTIL score of 10%) was also a prognostic marker for OS (*P =* 0.05) in the total patient cohort. PD-L1 positivity on immune cells predicted pCR after chemotherapy but no beneficial prognostic effect. All significant clinicopathological variables were included in a multivariate cox proportional hazards model (Table 3). In the total IBC cohort, only distant disease at diagnosis (HR 3.06, 95%CI 1.79–5.22, *P <* 0.001), sTIL infiltration (HR 0.46, 95%CI 0.27–0.81, *P =* 0.006) and nodal status (HR 1.64, 95%CI 1.14–2.35, *P =* 0.008) remained significant predictors for OS. In the group with initially localised disease, nodal disease and HR status were significantly associated with OS, and both pCR after NACT and HR status were associated with RFS (Table 3).

**Table 3:** Cox proportional hazards models for RFS, DMFS and OS. All significant clinicopathological variables were included in a multivariate Cox proportional hazards model. Distant disease at diagnosis, nodal status and sTIL infiltration are significantly associated with OS. In the group with initially localised disease, nodal disease was significantly associated with OS and both pCR after NACT and HR status were associated with RFS. *Significant *P* values in italic

Parameter	Hazard ratio	Lower 95% CI	Higher 95% CI	*P* value*
Cox proportional hazards model for OS in the total population				
sTIL (> 10%)	0.465	0.266	0.811	*0.006*
cN stage	1.635	1.137	2.353	*0.008*
cM stage	3.060	1.794	5.219	*< 0.001*
HR status	0.631	0.357	1.114	0.11
Cox proportional hazards model for RFS (initially localised disease)				
cN stage	1.354	0.876	2.093	0.17
HR status	0.471	0.249	0.889	*0.02*
Taxane NACT	0.934	0.424	2.055	0.86
pCR	0.406	0.166	0.992	*0.05*
Cox proportional hazards model for DMFS (initially localised disease)				
cN stage	1.530	0.981	2.39	0.06
HR status	0.564	0.295	1.08	0.08
Taxane NACT	0.771	0.348	1.71	0.52
pCR	0.391	0.149	1.03	0.06
Cox proportional hazards model for OS (initially localised disease)				
cN stage	1.652	1.020	2.674	*0.04*
HR status	0.453	0.224	0.918	*0.03*
Taxane NACT	0.672	0.296	1.524	0.34
pCR	0.368	0.126	1.075	0.07

### Validation cohort

To independently test our findings, we analysed an additional group of 64 IBC patients with non-metastatic disease, displaying similar clinicopathological characteristics as the discovery cohort (Additional file 1: Table S6 and Figure S6). Most of these patients received NACT followed by a mastectomy (59/64) and 28.8% (17/59) of the patients achieved pCR. Mean sTIL infiltration was 18.5% (95%CI: 14.7%–22.2%) and correlated with PD-L1 immunoreactivity (*P* < 0.001), comparable to the discovery cohort. Furthermore, sTIL were significantly (*P* = 0.05) higher in HR-negative IBC (median 22.5%) versus HR-positive IBC (median 10.0%). Tissue samples with more than 1% PD-L1+ tumour cells were not observed, but 38.7% (24/62) of the samples showed more than 1% PD-L1+ immune cells. An association between PD-L1 expression and clinicopathological features was not found, and PD-L1 immunoreactivity did not significantly correlate with pCR. However, 52.9% of the patients with pCR showed PD-L1 immunoreactivity vs. 32.5% of the patients without pCR (Additional file 1: Figure S7). A negative HR status (*P =* 0.04) and higher sTIL score (*P =* 0.01) correlated with pCR. However, in a logistic multivariate model, only sTIL score remained significant (OR 1.24, 95%CI 1.04–1.47, *P =* 0.02). The median OS was 8.86 years (CI 4.69–/ years), but no clinicopathological parameters, including sTIL score, were associated with a better OS or DFS (Additional file 1: Figure S8).

## Discussion

PD-L1 expression and sTIL infiltration were retrospectively analysed in a cohort of patients with IBC. We confirm the higher frequency of negative HR status in IBC tumours as compared to nIBC [23]. Almost half of the IBC patients (44.2%) presented with a HR-negative phenotype, while only 12% of all newly diagnosed breast cancers were HR-negative in our hospital (between 2010 and 2014) and SEER data reported that around 21% of the breast cancer patients have a HR-negative tumour type [24]. The lack of expression of hormone receptors has also been associated with a more aggressive clinical course in IBC [25]. In our study, patients with HR-positive tumours had a better RFS (HR 0.47, 95% CI 0.25–0.89, *P =* 0.02). Likewise, we confirmed the increased incidence of HER2 overexpression among IBC tumours [26]. However, in contrast to a large case-only analysis of 2014 women with IBC [27], we could not demonstrate any prognostic difference between patients with HER2-positive disease as compared to patients with HER2-negative disease. The reason for this difference is probably the lack of trastuzumab therapy in the majority of HER2-positive patients in our cohort treated before 2005, since trastuzumab therapy did predict a better OS in HER2-positive patients (*P =* 0.04).

Patients that received taxane-based NACT had a more favourable outcome in our cohort, and this confirms the findings made earlier by Cristofanilli et al [28]. We also confirm that patients with pCR after NACT have an improved outcome. However, despite the aggressive treatment, survival outcome is very poor with a 5-year OS of only 45.6%, similar to those reported by others [29, 30]. Additionally, relapse (either locally or distant metastasis) typically occurred early during follow-up with a median DFS of 3.5 years and brain-only relapse was seen in 17.3% of the patients. These data show that IBC is a clinically aggressive disease and that the underlying molecular determinants of the IBC phenotype will require more investigation.

Although breast cancer is considered moderately immunogenic, the presence of neo-antigens seems to elicit an immune response and infiltrating immune cells play an essential role in the host-defence mechanism against ER-negative, more proliferative subtypes of nIBC in both adjuvant and neo-adjuvant studies [31, 32]. In our IBC cohort, sTIL infiltration was higher in triple-negative IBC compared to other subtypes. The association between pCR and sTIL was only significant in the validation cohort. Using 142 nIBC controls, we also showed that the number of sTIL is comparable between IBC and nIBC. In the HER2+ subtype, the number of sTIL was significantly higher in the nIBC cohort; however, the median number of sTIL in IBC (luminal 10.0%, HER2+ 12.5%, TN 17.5%) was comparable with that reported in literature while the median of 23.75% for the HER2+ nIBC group was above what was expected [33]. Finally, sTIL infiltration was the only significant predictor of OS (HR 0.47, *P =* 0.024), besides nodal status and distant disease, in the multivariate model. Thus, it seems that in both IBC and (proliferative subtypes of) nIBC, immune cells play a crucial role in the long-term outcome of chemotherapy-treated patients. A hypothesis supported by the 107-gene signature, enriched for immunity-related genes that was able to distinguish between responders and non-responders to NACT in both an IBC and a nIBC group [7]. Furthermore, the expression of an immunomodulatory gene expression signature that indicates the presence of TILs in TNBC was not associated with IBC status [34]. Differences in IBC and nIBC might therefore be found in a different composition of the infiltrate or an altered function of the immune cells (e.g. by the expression of PD-L1).

To examine this, we stained 5-μm FFPE slides with a validated PD-L1 (Clone SP142, Ventana) assay. While this assay is sensitive for immune cell staining, less tumour cell staining is seen when compared to others [21, 35]. Nevertheless, we suggest that PD-L1 positivity using the SP142 antibody is clinically relevant as has been shown in patients with metastatic TNBC (mTNBC). The Impassion130 trial, using the same assay, showed that Atezolizumab plus paclitaxel prolonged survival in patients with mTNBC and that this benefit was larger in PD-L1+ patients [36]. A recent meta-analysis of predominantly early-stage breast cancer studies showed that PD-L1 positivity (expression on the tumour and/or immune cells) was associated with a worse OS (HR 1.76, 95%CI 1.09–2.82, *P =* 0.02) and adverse clinicopathological features [11]. Interestingly, PD-L1 mRNA positivity was associated with a better prognosis and response to therapy in TNBC [12]. A better outcome was also observed in TNBC patients with PD-L1 expression on the infiltrating immune cells [13, 37]. This might indicate that the prognostic effect of PD-L1 depends on the underlying (subtype-specific) immune response. Data on PD-L1 expression in IBC are limited (Table 1). Furthermore, there are substantial differences in study population, sample type and used assays (used AB, scoring method, cutoff values, immune or tumour cell). This renders comparison of PD-L1 expression between different studies unreliable.

Immune cell positivity for PD-L1 was seen in 42.9% of the IBC patients, which is higher than previous studies have reported in unselected patients with breast cancer (6.0%) [12] and in patients with TNBC (31.6%) [32]. Although our results are in agreement with the high PD-L1 positivity rates that were observed in other IBC studies [16, 17], we are the first to demonstrate that, independent of molecular subtype, PD-L1 expression in IBC is more frequent than in nIBC (OR 2.43, *P =* 0.01). In a validation cohort, we showed a similar PD-L1 expression (38.6%, *P =* 0.8, Additional file 1: Table S5) that was also more frequent than in nIBC (*P =* 0.04).

**Fig. 5:**
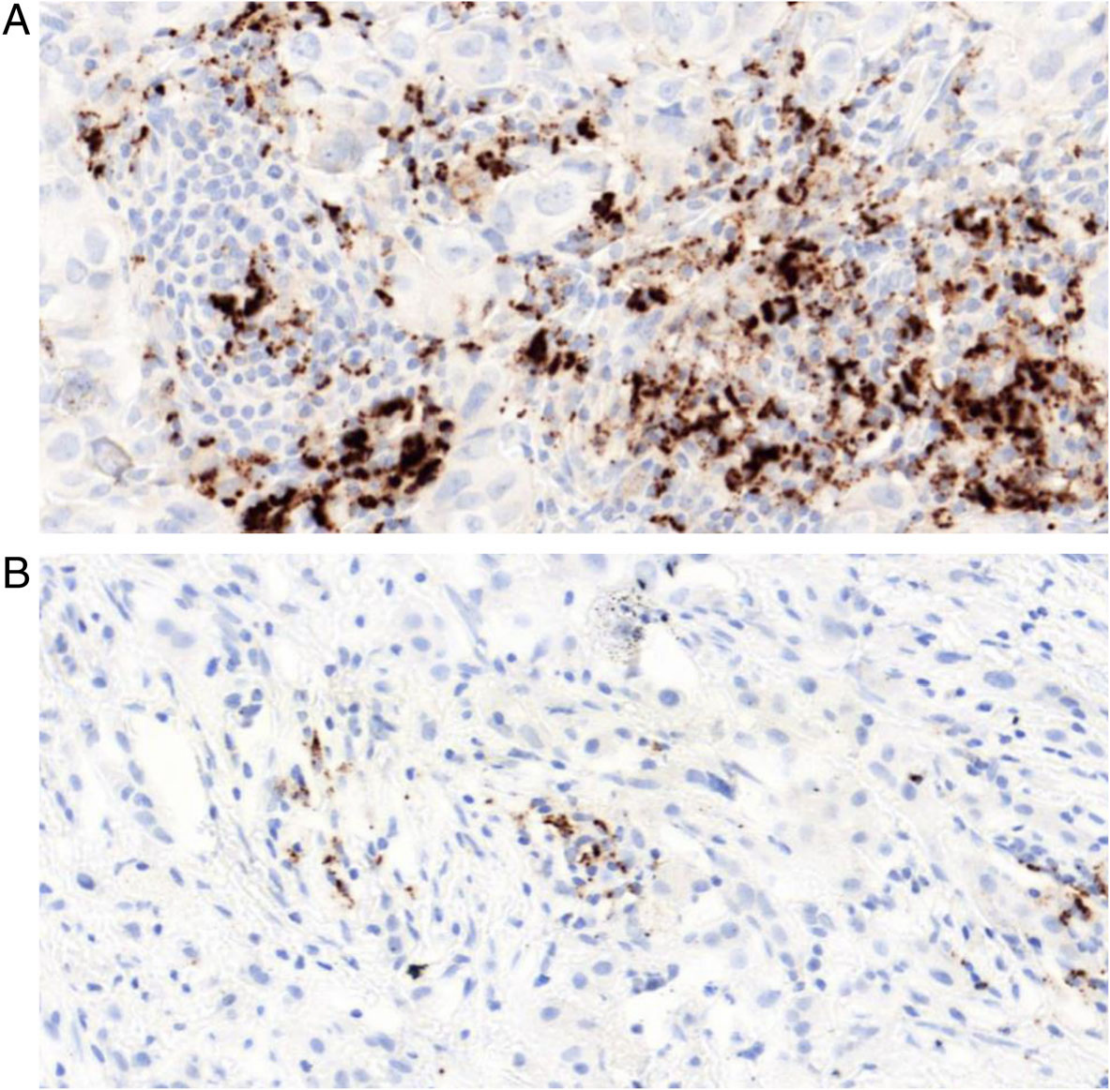
PD-L1+ immune cells (AB: SP142, Brown DAB staining) in IBC (× 250). There is a strong association between PD-L1 and sTIL, without PD-L1 expression on the tumour cells. This suggests an adaptive expression pattern. **a** High PD-L1 expression (> 10%, category 3). **b** Low PD-L1 expression (> 1% and < 5%, category 1)

Some studies have demonstrated a positive relationship between PD-L1 and a cytotoxic T cell response [15] or CD20+ TILs [16]. In our cohorts, there also was a strong correlation with sTIL score, suggesting that PD-L1 expression is related to immune cell activation (Fig. 5). Together with the frequent expression, this adaptive expression pattern of PD-L1 suggests a suppressed immune response phenotype in IBC. Immune checkpoint modulators might therefore be useful in the treatment of IBC patients.

Similar to the study of Arias-Pulido et al., the level of tumour staining was low in our cohorts [16]. This can be related to the used antibody, but a low constitutive expression of PD-L1 on IBC tumour cells might be another explanation. We could not confirm the reported association between PD-L1 and grade or other clinicopathological features [16]. There are however substantial clinical differences between both studies. Most importantly, populations with a different genetic background with different proportions of molecular subtypes are being analysed. The different scoring methodology and the use of a TMA might also have contributed to these discrepancies. At present, the prognostic role of PD-L1 expression in IBC remains unclear. We could not corroborate a beneficial effect of PD-L1 positivity, and others have even reported a worse OS in IBC patients with PD-L1-positive tumour cells [17]. Finally, we confirm a positive correlation between PD-L1 expression and pCR after NACT [16].

The strength of this study is the analysis of a large cohort and a validation cohort of patients with a rare form of breast cancer. We managed to explore disease characteristics, clinical evolution and immune parameters of IBC over a long period of time (1996–2018). PD-L1 antigenicity decreases over time, a problem inherent to all retrospective IHC studies. Although PD-L1 expression was reduced in the older FFPE blocks, the year of diagnosis did not affect PD-L1 expression on the sTIL in a multivariate model (Additional file 1: Figure S9). Other potential limitations of this study are its retrospective character, the double-centre design and the fact that there are some missing or incomplete data.

## Conclusions

IBC is characterised by a higher frequency of more proliferative, HR-negative tumours. Similar to nIBC, patients with a HR-positive tumour have a better prognosis. PD-L1 immunoreactivity on immune cells was seen in 42.9% of our patients and in 38.6% of the patients in the validation cohort. This was significantly higher than the nIBC control group and suggests a possible role for immune checkpoint inhibitors in the treatment of IBC. Infiltration with lymphocytes (sTIL) was a significant predictor of OS (HR 0.47, 95%CI 0.27–0.81, *P =* 0.006). The prognostic role of sTIL, together with the high frequency of PD-L1-positive immune cells, is an indication that an activated but suppressed immune microenvironment contributes to the aggressive and unique biological features associated with IBC.

## Additional file

Additional file 1: **Table S1.** Minimum criteria required for the diagnosis of IBC. **Table S2.** Correlation between PD-L1 and clinicopathological tumour characteristics. **Table S3.** A logistic multivariate model for PD-L1 expression on immune cells. **Table S4.** Patient and tumour characteristics of the nIBC control group. **Table S5.** Frequencies of organ-specific metastases. **Table S6.** Patient and tumour characteristics of the IBC validation cohort from Marseille. **Figure S1.** Neo-adjuvant chemotherapy regimens. **Figure S2.** Kaplan-Meier curves of RFS and OS in the total IBC population (143 patients). **Figure S3.** Kaplan-Meier curves of significant prognostic variables for RFS. **Figure S4.** Kaplan-Meier curves of significant prognostic variables for DMFS. **Figure S5.** Kaplan-Meier curve showing significant survival benefit for HER2-positive IBC patients that received trastuzumab. **Figure S6.** Clinicopathological characteristics of the validation cohort. **Figure S7.** PD-L1 immunoreactivity and pCR in the validation cohort. **Figure S8.** Prognostic clinicopathological variables in the validation cohort. **Figure S9.** PD-L1 antigenicity decreases over time. (DOCX 10547 kb)

## Abbreviations

DMFS: Distant metastasis-free survival
ER: Estrogen receptor
FFPE: Formalin-fixed paraffin-embedded
H&E: Haematoxylin and eosin
HR-negative: Hormone receptor-negative
HR-positive: Hormone receptor-positive
IBC: Inflammatory breast cancer
ICC: Interclass correlation coefficient (ICC)
mTNBC: Metastatic triple-negative breast cancer
NACT: Neo-adjuvant chemotherapy
nIBC: Non-inflammatory breast cancer
OS: Overall survival
pCR: Complete pathologic response
PD-L1: Programmed death-ligand 1
PgR: Progesteron receptor
RFS: Recurrence-free survival
sTIL: Stromal tumour-infiltrating lymphocytes
TILs: Tumour-infiltrating lymphocytes
TMA: Tissue microarray
TNBC: Triple-negative breast cancer
WIBCC: World IBC consortium
