# Supplementary material for: Infiltrating stromal immune cells in inflammatory breast cancer are associated with an improved outcome and increased PD-L1 expression
Source: Breast Cancer Res. 2019 Feb 18;21:28. doi: 10.1186/s13058-019-1108-1 (PMC6380068; doi:10.1186/s13058-019-1108-1)
Supplement: Supplementary file 1 — Table S1. Minimum criteria required for the diagnosis of IBC. Table S2. Correlation between PD-L1 and clinicopathological tumour characteristics. Table S3. A logistic multivariate model for PD-L1 expression on immune cells. Table S4. Patient and tumour characteristics of the nIBC control group. Table S5. Frequencies of organ-specific metastases. Table S6. Patient and tumour characteristics of the IBC validation cohort from Marseille. Figure S1. Neo-adjuvant chemotherapy regimens. Figure S2. Kaplan-Meier curves of RFS and OS in the total IBC population (143 patients). Figure S3. Kaplan-Meier curves of significant prognostic variables for RFS. Figure S4. Kaplan-Meier curves of significant prognostic variables for DMFS. Figure S5. Kaplan-Meier curve showing significant survival benefit for HER2-positive IBC patients that received trastuzumab. Figure S6. Clinicopathological characteristics of the validation cohort. Figure S7. PD-L1 immunoreactivity and pCR in the validation cohort. Figure S8. Prognostic clinicopathological variables in the validation cohort. Figure S9. PD-L1 antigenicity decreases over time. (DOCX 10547 kb) [file 13058_2019_1108_MOESM1_ESM.docx]

**Additional file 1**

| 1. Rapid onset of breast erythema, edema and/or peau d’orange, and/or warm breast, with or without an underlying palpable mass. |
| --- |
| 2. Duration of history of no more than 6 months. |
| 3. Erythema occupying at least one-third of the breast. |
| 4. Pathologic confirmation of invasive breast carcinoma. |

Table S1 Minimum criteria required for the diagnosis of IBC

|  | | **PD-L1-  sTIL** | **PD-L1+**  **sTIL** | ***P-* value**  **(χ^2^ test)** | ***P-* value**  **(GLM)** |
| --- | --- | --- | --- | --- | --- |
| cN-stage | 0 | 5 | 1 | 0.6 |  |
|  | 1 | 37 | 16 |  |  |
|  | 2 | 36 | 16 |  |  |
|  | 3 | 17 | 12 |  |  |
| cM-stage | 0 | 70 | 33 | 1 |  |
|  | 1 | 28 | 12 |  |  |
| Pathological type | Ductal | 90 | 44 | 0.4 |  |
|  | Lobular | 5 | 0 |  |  |
|  | Mixed | 2 | 1 |  |  |
| Differentiation | Grade 1 | 3 | 0 | **0.01** |  |
|  | Grade 2 | 30 | 5 |  | 0.75 |
|  | Grade 3 | 57 | 38 |  | 0.44 |
| ER | Negative | 41 | 26 | 0.1 |  |
|  | Positive | 55 | 19 |  |  |
| PgR | Negative | 51 | 26 | 0.6 |  |
|  | Positive | 43 | 19 |  |  |
| HER2+ | Negative | 38 | 25 | 0.8 |  |
|  | Positive | 58 | 20 |  |  |
| Molecular subtype | HR+ | 56 | 20 | 0.2 |  |
|  | HR-HER2+ | 19 | 11 |  |  |
|  | HR-HER2- | 18 | 14 |  |  |
| sTIL | < 10% | 31 | 7 | **<0.001** |  |
|  | >=10 – < 40% | 27 | 28 |  | **0.02** |
|  | >= 40 % | 3 | 10 |  | **0.001** |

**Table S2** Correlation between PD-L1 and clinicopathological tumour characteristics. *P-* Values for the PD-L1+ sTIL group compared to the PD-L1- sTIL group. (Pearson Chi^2^ test). In a multivariate model, only the strong correlation between PD-L1 positivity and sTIL scores remained significant.

| **A. Logistic multivariate model for pCR after NACT** | | | | |
| --- | --- | --- | --- | --- |
| Parameter | Odds ratio | Lower 95% CI | Higher 95% CI | P-value |
| sTIL score | 1.00 | 0.99 | 1.01 | 0.74 |
| PD-L1 (IC) | 1.22 | 1.07 | 1.40 | **0.005** |
| (Intercept) | 1.10 | 0.942 | 1.27 | 0.24 |
| **B. Logistic multivariate model with additional significant prognostic parameters** | | | | |
| Parameter | Odds ratio | Lower 95% CI | Higher 95% CI | P-value |
| (Intercept) | 1.00 | 0.67 | 1.52 | 0.98 |
| cN stage | 0.92 | 0.80 | 1.06 | 0.24 |
| HR status | 1.02 | 0.82 | 1.26 | 0.86 |
| sTIL score | 1.00 | 0.99 | 1.01 | 0.70 |
| Taxane therapy | 1.27 | 0.89 | 1.79 | 0.18 |
| PD-L1 (IC) | 1.22 | 1.06 | 1.40 | **0.007** |

**Table S3** **A.** In a logistic multivariate model, PD-L1 expression on IC remained significant for predicting pCR. **B.** In a logistic multivariate model with the addition of cN stage, HR status and sTIL score (the 3 prognostic parameters for patients with initially localized disease): PD-L1 expression remained the only significant predictor for pCR after NACT.

|  |  | | *P-* Value (vs IBC) |
| --- | --- | --- | --- |
| Mean age | 58.4 y (30.0 y –94.6 y) | | = .30 |
| Mean sTIL score | 22.3%, 95% CI: 18.6 %– 25.94% | | = .07 |
| Menopausal status | Premenopausal | 23.9 %) | = .44 |
|  | Postmenopausal | 76.1 %) |  |
| **cN-stage** | 0 | 61.4% | **< .001** |
|  | 1 | 23.9% |  |
|  | 2 | 11.4% |  |
|  | 3 | 3.4% |  |
| **cM-stage** | 0 | 100% | **< .001** |
|  | 1 | 0.0% |  |
| APO | Ductal | 94.3% | = .49 |
|  | Lobular | 5.7% |  |
|  | Mixed | 0.0% |  |
| Differentiation | Grade 1 | 8.0% | =.13 |
|  | Grade 2 | 23.9% |  |
|  | Grade 3 | 68.2% |  |
| HR | Negative | 40.4% | =.53 |
|  | Positive | 59.6% |  |
| HER2+ | Negative | 61.7% | =.38 |
|  | Positive | 38.2% |  |
| Molecular subtype | Luminal | 57.0% | =.91 |
|  | HER2+ | 18.3% |  |
|  | TN | 24.6% |  |
| sTIL | < 10% | 31.0% | =.23 |
|  | >=10 – < 40% | 48.6% |  |
|  | >= 40 % | 20.4% |  |
| **PD-L1 immune cells** | < 1% | 76.3% | **=.006** |
|  | >= 1 % - <5% | 18.7% |  |
|  | >= 5% - < 10% | 3.6% |  |
|  | >= 10% | 1.4% |  |

**Table S4** Patient and tumor characteristics of the nIBC control group. *P-* Values for the nIBC patients compared to the IBC cohort (Pearson Chi^2^ test, ANOVA for mean age and sTIL score)

|  | **Lung** | | **Liver** | | **Bone** | | **Brain** | |
| --- | --- | --- | --- | --- | --- | --- | --- | --- |
|  | M- (63/92)  68.5 % | M+  (29/92)  31.5% | M-  (25/92)  27.1% | M+  (57/92)  61.9% | M-  (50/92)  54.3% | M+  (42/92)  45.7% | M-  (59/92)  64.1% | M+  (33/92)  35.9% |
| **cM0 (52/92)**  **56.5 %** | 37 | 15 | 26 | 26 | 30 | 22 | 31 | 21 |
| **cM1 (40/92)**  **43.5 %** | 26 | 14 | 9 | 31 | 20 | 20 | 28 | 12 |

**Table S5** Frequencies of organ-specific metastases of all patients that developed distant disease: 56,5% of the patients developed metastases during therapy or follow-up (M0) while 43.5% of the patients already had distant metastasis at the moment of diagnosis (M1). M-: No metastasis, M+: Metastasis.

|  |  | | *P-* Value (vs IBC GZA) |
| --- | --- | --- | --- |
| APO (n= 64) | Ductal (n= 54) | 84.4% | 0.1 |
|  | Lobular (n= 7) | 11.0 % |  |
|  | Mixed (n= 3) | 4.7 % |  |
| Differentiation (n= 61) | Grade 1 (n= 5) | 8.2 % | 0.07 |
|  | Grade 2 (n= 15) | 24.6 % |  |
|  | Grade 3 (n= 41) | 67.2 % |  |
| ER (n= 64) | Negative (n= 26) | 40.6% | 0.4 |
|  | Positive (n= 38) | 59.4 % |  |
| PgR (n= 64) | Negative (n= 31) | 48.4 % | 0.08 |
|  | Positive (n= 33) | 51.6 % |  |
| HR (n= 64) | Negative (n= 25) | 39.1 % | 0.5 |
|  | Positive (n= 39) | 60.9 % |  |
| HER2+ (n= 52) | Negative (n= 34) | 65.4 % | 0.2 |
|  | Positive (n= 18) | 34.6 % |  |
| Molecular subtype (n= 52) | Luminal (n= 31) | 59.6 % | 0.2 |
|  | HER2+ (n= 9) | 17.3 % |  |
|  | TN (n= 12) | 23.1 % |  |
| sTIL (n= 64) | < 10% (n= 21) | 32.8 % | 0.9 |
|  | >=10 – < 40% (n= 34) | 53.1 % |  |
|  | >= 40 % (n= 9) | 14.0 % |  |
| PD-L1 immune cells (n= 62) | < 1% (n= 38) | 61.3 % | 0.8 |
|  | >= 1 % - <5% (n= 16) | 25.8 % |  |
|  | >= 5% - < 10% (n= 7) | 11.2 % |  |
|  | >= 10% (n= 1) | 1.6 % |  |
| Mean sTIL score | 18.5 %, 95% CI: 14.7 %– 22.2 % | | 0.8 |

**Table S6** Patient and tumor characteristics of the IBC Validation Cohort from Marseille. (n) = number of patients. *P-* Values for the IBC patients from Antwerp compared to the IBC cohort from Marseille (Pearson Chi^2^ test, ANOVA for mean sTIL score) show no significant differences between both IBC populations.

A.
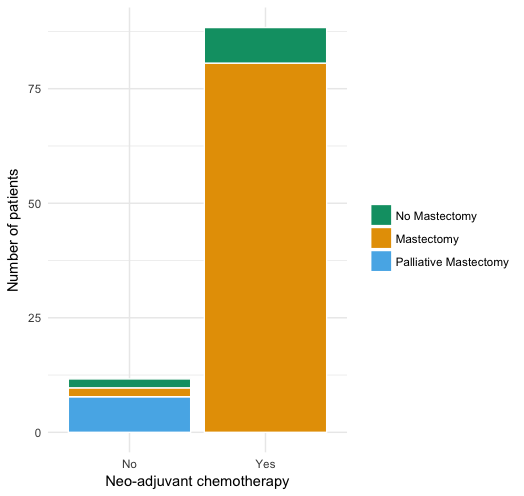


B.
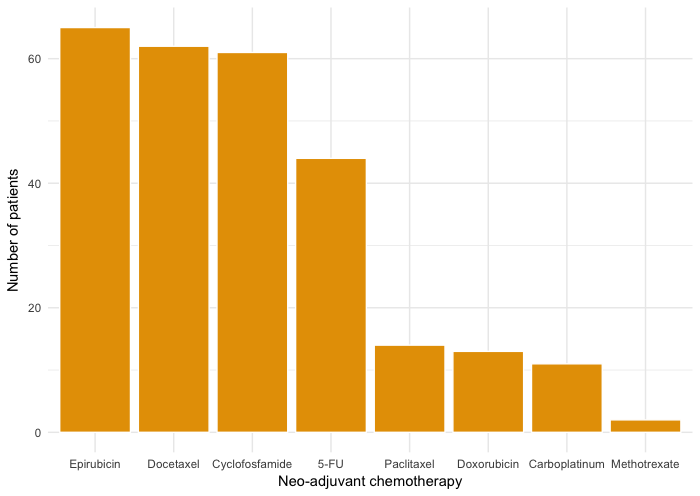


**Figure S1** Neo-adjuvant chemotherapy **A.** 91 out of 103 patients without metastatic disease on diagnosis (88.3%) completed neo-adjuvant chemotherapy. 83 patients underwent a subsequent mastectomy (91.2%). **B.** An anthracycline based regime (Epirubicin or Doxorubicin, n=78/91, 85.7%) combined with a taxane (Docetaxel or Paclitaxel, n=74/91, 81.3%) was the most common type of NACT.


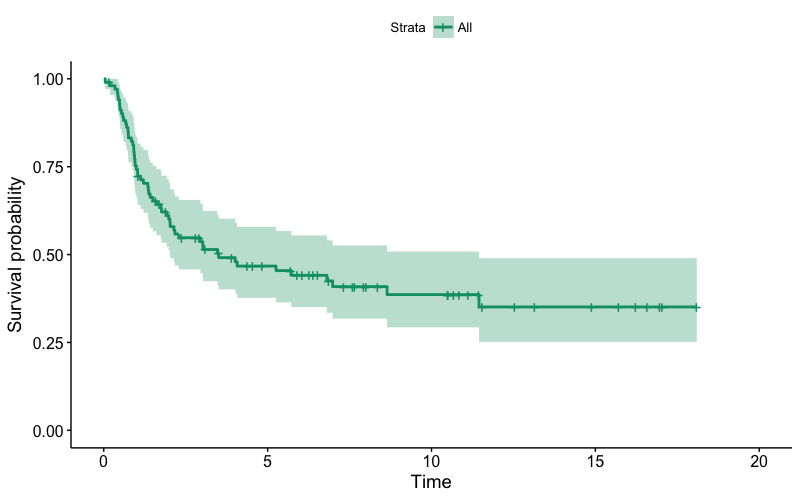


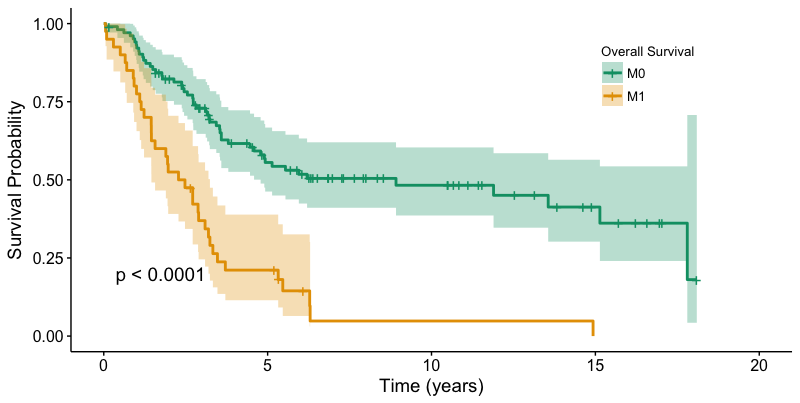


**Figure S2** **A.** RFS: After 5 years, more than half of the patients (53.3%) had relapsed. **B.** Overall survival in the total IBC population (143 patients): Patients with initially localized disease live significantly longer than patients with primary metastatic disease (Median OS: 8.92 years vs. 2.38 years, p < 0.0001)

A.
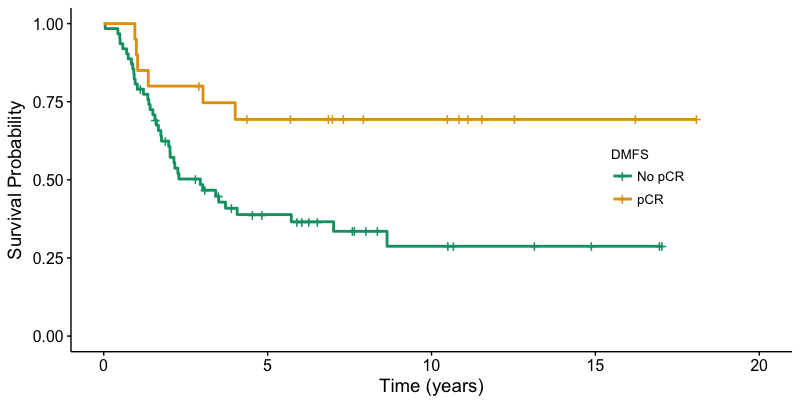


**χ^2^ = 6.1, *P=* 0.01**

**5y DMFS: 38.4 % vs. 69.3%**

B.
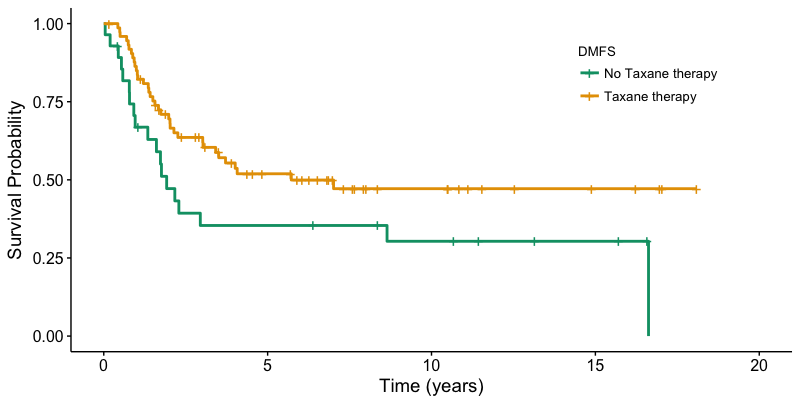


**χ^2^ = 4.4-, *P=* 0.03**

**5y DMFS: 35.4% vs. 51.9%**

C.
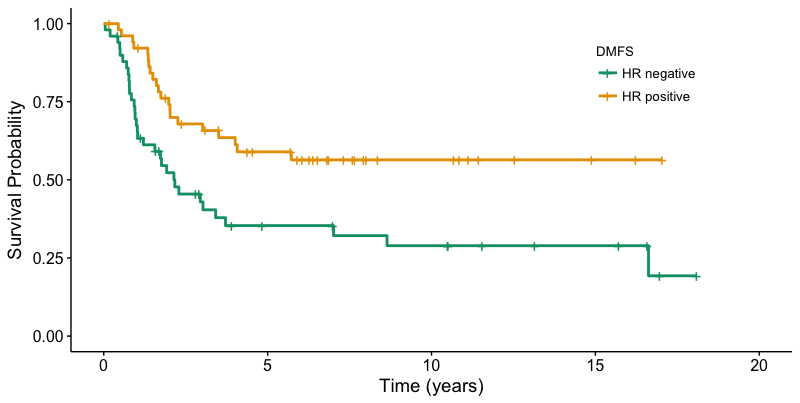


**χ^2^ = 8.3, *P=* 0.004**

**5y DMFS: 35.4% vs. 59.0%**

D.
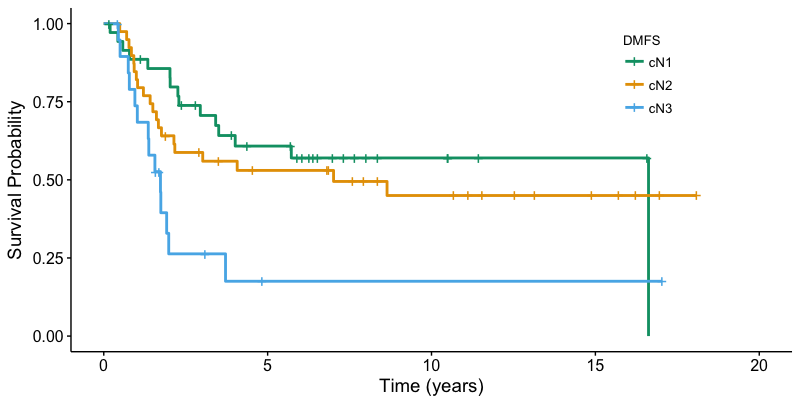


**χ^2^ = 9.8, *P=* 0.007**

**5y DMFS: 17.5% vs. 53.0% vs. 60.8%**

**χ^2^ = 5, *P=* 0.02**

**5y DMFS: 27.8% vs. 51.2%**

**Figure S3** Kaplan Meier curves of significant prognostic variables for RFS: **A.** pCR after NACT **B**. Taxane containing chemotherapy, **C.** Hormone receptor status and **D.** Nodal status

A.
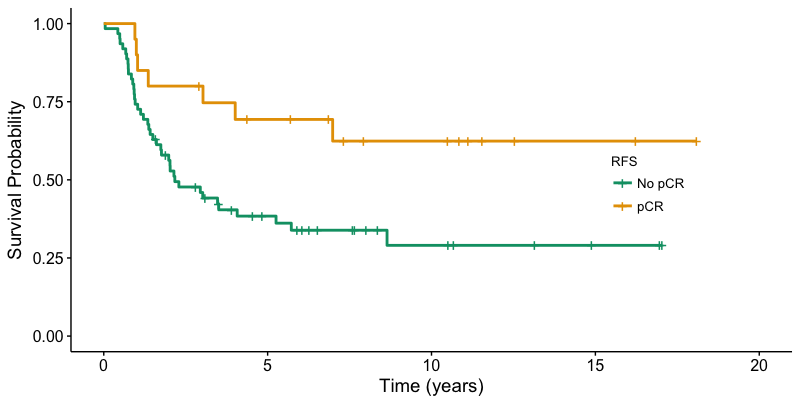


**χ^2^ = 6.1, *P=* 0.01**

**5y RFS: 38.4% vs. 69.3%**

B.
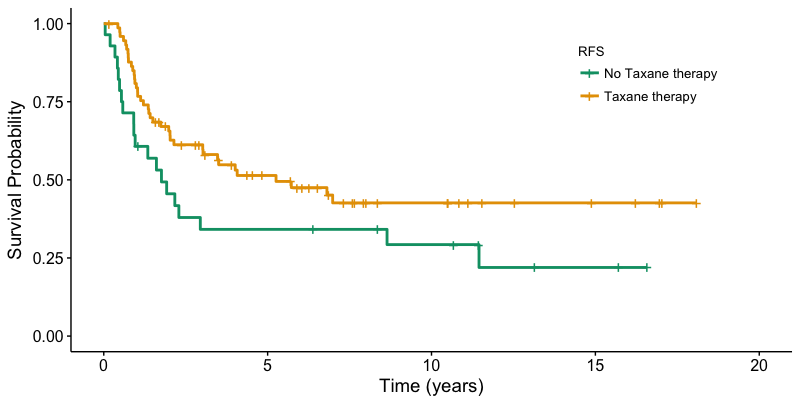


**χ^2^ = 3.8, *P=* 0.05**

**5y RFS: 34.2% vs. 51.4%**

C.
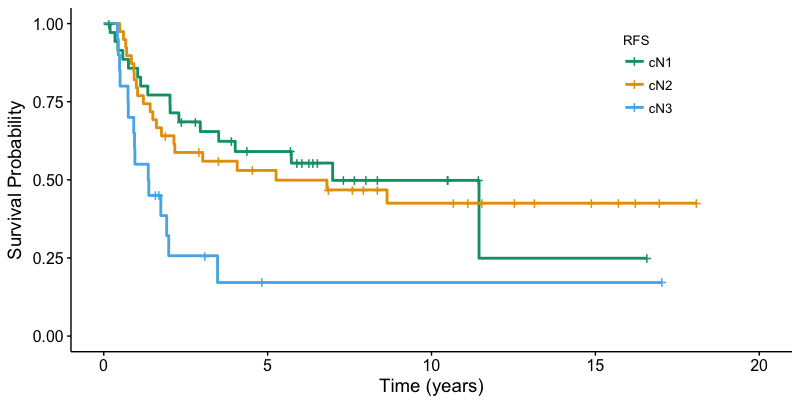


**χ^2^ = 10.4, *P=* 0.005**

**5y RFS: 17.1% vs. 53.0% vs. 59.1%**

D.
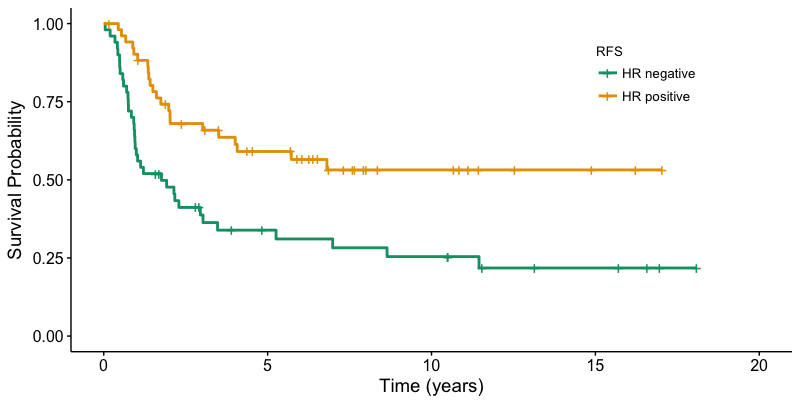
**Figure S4** Kaplan Meier curves of significant prognostic variables for DMFS: **A.** pCR after NACT, **B.** Taxane containing chemotherapy, **C.** Nodal status and **D.** HR status

**χ^2^ = 10.5, *P=* 0.001**

**5y RFS: 33.9% vs. 59.1%**


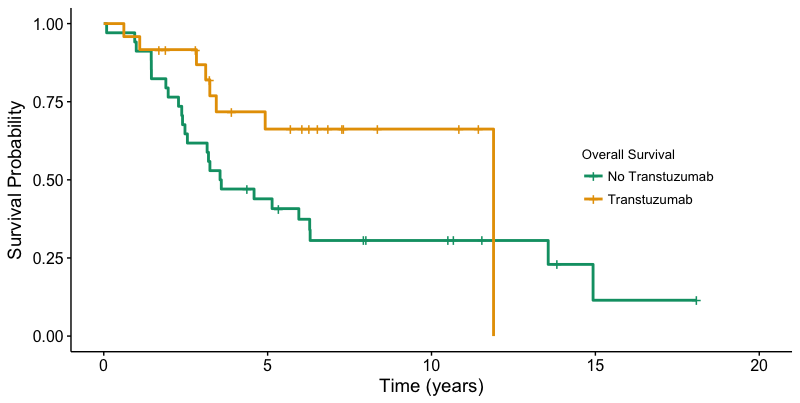


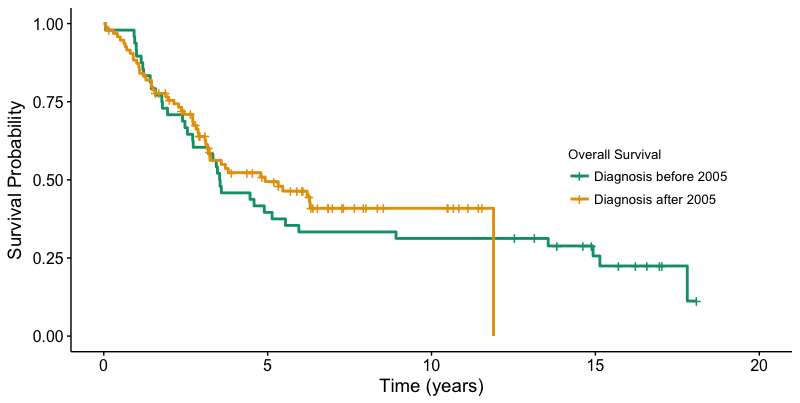


**Figure S5** **A**. Kaplan Meier curve showing significant survival benefit for HER2 positive IBC patients that received trastuzumab. 5y OS: 66.2% vs 43.9%, χ^2^ = 4.11, *P=* 0.042 **B**. Kaplan Meier curves showing no significant survival difference between patients diagnosed before or after 31/12/2005, when trastuzumab was added to the treatment of HER2 positive IBC. *P=* 0.50.

1.
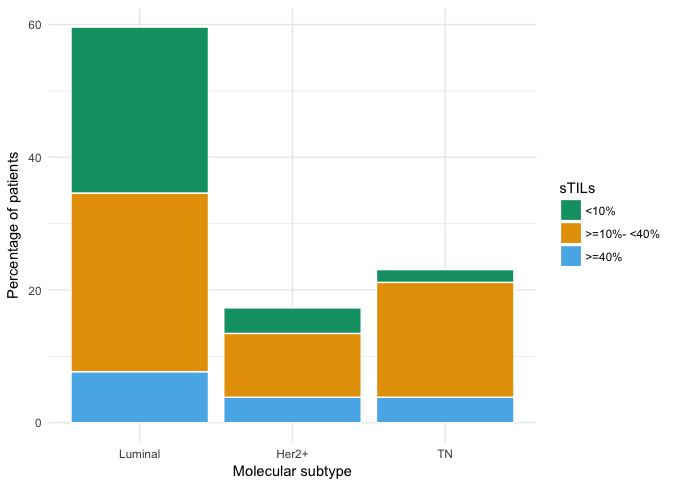

2.
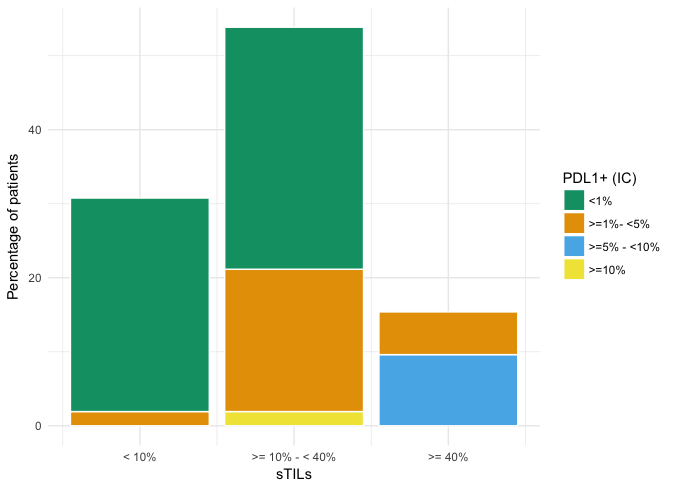


**Figure S6** Clinicopathological characteristics of the validation cohort: **A**. Distribution of the molecular subtypes (n= 52). Luminal (HR+): 59.6% (n= 31), HER2+: 17.3% (n= 9), TN: 23.4 % (n= 12). **B.** Distribution of the stromal TIL scores (n= 64); respectively 32.8 %(n= 21), 53.1 % (n= 34), 14.0 % (n= 9). There is a strong correlation with PD-L1 immunoreactivity on immune cells (χ2 = 43.9, *P<* 0.001)


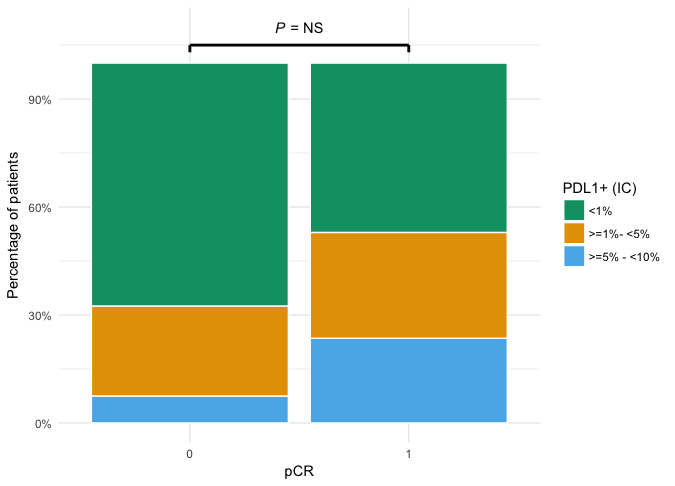


**Figure S7** Patients that had a complete pathological response (17/57) showed more PD-L1 immunoreactivity (52.9% PD-L1+ IC) than patients without a complete response (40/57, 32.5% PD-L1+ IC), although this did not reach statistical significance in the validation cohort. χ^2^ = 3.4, *P=* 0.18.


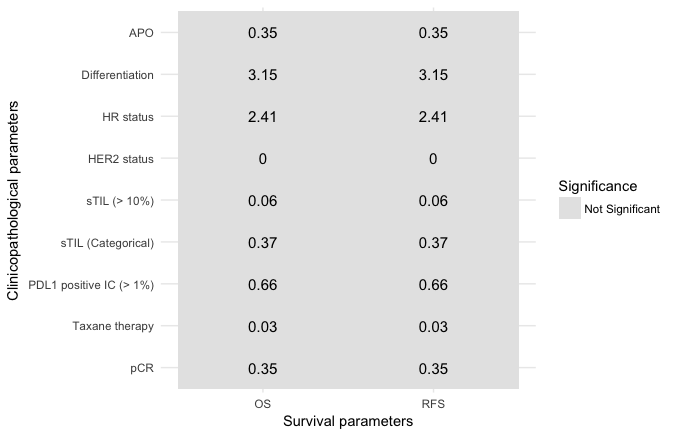


**Figure S8** An overview of all clinicopathological values and the χ^2^ value in the tables shows that in the validation cohort no variable had prognostic significance for OS or DFS.


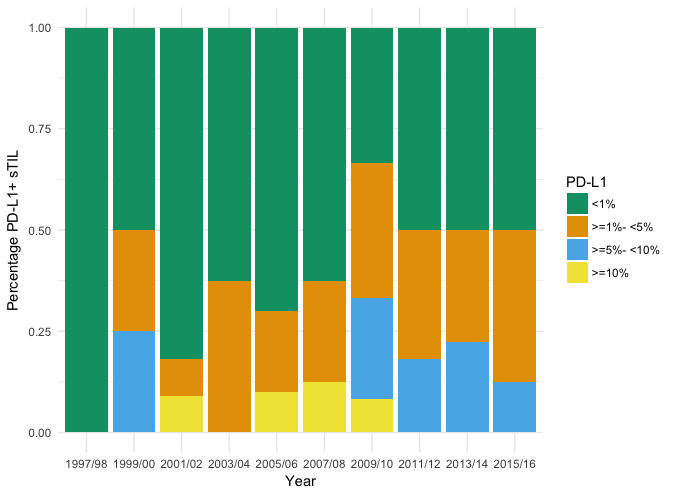


**Figure S9** PD-L1 antigenicity decreases over time. There was a correlation between year of diagnosis and PD-L1 positivity on immune cells (*P=* 0.03). However, in a multivariate model, with sTIL score and differentiation, this effect was not significant anymore (*P=* 0.08).
